# Thanks to the Editorial Board of *Arquivos de Neuro-Psiquiatria* (2024)

**DOI:** 10.1055/s-0045-1802322

**Published:** 2025-01-31

The Editors of *Arquivos de Neuro-Psiquiatria* extend their sincere gratitude to the **Deputy Editors** (2), **Associate Editors** (80), **Editorial Board Members** (52), and **Referees** (362) for their altruistic and unpaid dedication to advancing the editorial tasks of this journal from January 1, 2024 to December 31, 2024.

Thank you very much!

**DEPUTY EDITORS**

Carlos Henrique Ferreira Camargo

Clécio de Oliveira Godeiro Junior

**ASSOCIATE EDITORS**

Adriana Bastos Conforto

Alex Tiburtino Meira

Alexandra Prufer Queiroz Campos Araújo

Aline Chacon Pereira

Ana Carolina Coan

Analuiza Camozzato

Antonio Arauz Góngora

Antonio José da Rocha

Antonio Lucio Teixeira

Caio César Diniz Disserol

Carlos Otto Heise

Carolina de Medeiros Rimkus

Chien Hsin Fen

Clarissa Lin Yasuda

Cristiane Nascimento Soares

Diana Aguiar de Sousa

Douglas Kazutoshi Sato

Edmar Zanoteli

Eduardo Genaro Mutarelli

Eliasz Engelhardt

Elisa de Paula França Resende

Ethel Mizrahy Cuperschmid

Eva Carolina Rocha

Fabíola Dach

Fernando Morgadinho Santos Coelho

Francisco de Assis Aquino Gondim

Francisco Manoel Branco Germiniani

Gabriel Rodriguez de Freitas

Gisele Sampaio Silva

Gustavo Leite Franklin

Iscia Teresinha Lopes Cendes

Jamary Oliveira Filho

João Brainer Clares de Andrade

José Luiz Pedroso

Juliana Gurgel-Giannetti

Karen Nunez-Wallace

Karina Gomes

Laura Silveira Moriyama

Lea Tenenholz Grinberg

Leandro Tavares Lucato

Lécio Figueira Pinto

Leonardo Cruz de Souza

Lívia Almeida Dutra

Luciano De Paola

Luciene Covolan

Luis Filipe de Souza Godoy

Luís Otávio Sales Ferreira Caboclo

Luiz Eduardo Gomes Garcia Betting

Marcondes Cavalcante França Jr

Marcos Christiano Lange

Maria Fernanda Mendes

Mariana Spitz

Marina Koutsodontis Machado Alvim

Mario Fernando Prieto Peres

Marzia Puccioni-Sohler

Mônica Sanches Yassuda

Octávio Marques Pontes Neto

Orlando Graziani Povoas Barsottini

Paulo José Lorenzoni

Paulo Pereira Christo

Pedro Andre Kowacs

Pedro Augusto Sampaio Rocha-Filho

Pedro Rosa-Neto

Rafaelle Ornello

Raffaele Ornello

Renato Puppi Munhoz

Rosana Hermínia Scola

Rosana Souza Cardoso Alves

Sarah Teixeira Camargos

Sérgio Monteiro de Almeida

Sérgio Rosemberg

Sheila Cristina Ouriques Martins

Sônia Maria Dozzi Brucki

Suzana Maria Fleury Malheiros

Sylvie Devalle

Tamine Teixeira da Costa Capato

Tarso Adoni

Vitor Tumas

Wilson Marques Jr.

Ylmar Correa Neto

**EDITORIAL ADVISORY BOARD**

Acary Souza Bulle Oliveira

Alberto J. Espay

Alexis Brice

Américo Ceiki Sakamoto

Andrea Slachevsky

Andrew J. Lees

Bruce Miller

Bruce Ovbiagele

Carlos Alberto Mantovani Guerreiro

Carlos Roberto de Mello Rieder

Christina Marra

Didier Leys

Fernando Cendes

Fernando Kok

Francisco Eduardo Costa Cardoso

Giancarlo Comi ( *in memoriam* )

Gilmar Fernandes do Prado

Henrique Ballalai Ferraz

Hugh J. Willison

Jaderson Costa da Costa

João José Freitas de Carvalho

Joaquim Ferreira

Joaquim Pereira Brasil Neto

José Manuel Ferro

Lineu César Werneck

Luiz Henrique Martins Castro

Márcia Lorena Fagundes Chaves

Marco Aurélio Lana-Peixoto

Marcos Raimundo Gomes de Freitas

Maria José Sá

Maria Lucia Brito Ferreira

Marilisa Mantovani Guerreiro

Mônica Levy Andersen

Oscar Del Brutto

Oscar Gershanik

Osvaldo José Moreira do Nascimento

Osvaldo Massaiti Takayanagui

Paulo Caramelli

Pedro Chaná-Cuevas

Raimundo Pereira da Silva Neto

Regina Maria Papais-Alvarenga

Ricardo Allegri

Ricardo Nitrini

Roger Walz

Rubens José Gagliardi

Sérgio Teixeira Ferreira

Simona Sacco

Stefan Schwab

Ugo Nocentini

Umbertina Conti Reed

Vladimir Hachinski

Walter Rocca

**REVIEWERS**

Abelardo Queiroz Campos Araújo

Acary Souza Bulle Oliveira

Adelia Maria Henriques-Souza

Adriana Carra

Adriana Ponsoni

Alex Meira

Alexandra Araújo

Alexandre Alamy

Alexandre Fernandes

Alfonso Fasano

Aline Mizuka Kozoroski Kanashiro

Aline Pereira

Aline Vitali da Silva

Alves Cesar

Amnuay Kleebayoon

Ana Carolina Coan

Ana Carolina Monteiro Lessa de Moura

Ana Claudia de Souza

Ana Cláudia Vieira

Ana Paula Etges

André Palmini

André Silva

Arlete Hilbig

Artur Schumacher-Schuh

Ayrton Roberto Massaro

Bárbara Panichelli

Barbosa Alcino

Beatriz Monteiro

Benedicto Oscar Colli

Bhagat Singh

Breno Barbosa

Bruno Pedreira

Bruno Rodrigues

Burhan Fatih Koçyiğit

Camila Aquino

Camila Pupe

Carlo Domênico Marrone

Carlos Augusto Takeuchi

Carlos Bernardo Tauil

Carlos Cosentino

Carlos Eduardo Soares Silvado

Carlos Eduardo Veloso

Carlos Henrique Camargo

Carlos Mauricio Almeida

Carlos Otto Heise

Carolina Candeias

Carolina de Medeiros Rimkus

Carolina Lavigne Moreira

Carolina Rouanet

Carolina Soares

Carolina Souza

Caroline Araújo

Cássia de Alcântara

Cassio Meira

Celiana Viana

Chong Kim

Christiane Campos

Clara Barreira

Clara Camelo

Clarice Listik

Clarisse Fortes

Cláudia Vasconcelos

Clécio Godeiro-Junior

Cleiton Formentin

Cleonisio Rodrigues

Conceicao Campanario

Cristiane Moreno

Cristiano Aguzzoli

Daiwai Olson

Daniel de Barros

Daniela Gil

Danielle Bruno

Darciane Baggjo

Dayany Boone

Débora Maia

Denis Bernardi Bichuetti

Denis Gabriel

Denise Maria Cury Portela

Dennis Schutter

Diana Melancia

Diego Fragoso

Dilan Demirtas Karaoba

Dilara Onan

Diogo Correa

Diogo Veiga

Dong Yan

Edmar Zanoteli

Eduardo Estephan

Eduardo Silva

Einstein Francisco de Camargos

Elen Beatriz Pinto

Eliana da Cunha

Eliasz Engelhardt

Elisa de Melo

Elisa Resende

Ellen Lima

Elmano Henrique Torres Carvalho

Emanuel Cassou

Erasmo Barbante Casella

Eric Anthony Sribnick

Erica Tardelli

Ethel Ciampi

Eva Rocha

Fabiana Orlandi

Fabio Barroso

Farmy Silva

Fei Peng

Felipe Oliveira

Felipe Pacheco

Felipe Reis

Felipe Saba

Felipe Sudo

Felipe von Glehn

Feres Chaddad-Neto

Fernando Cendes

Fernando Morgadinho Coelho

Fernando Stelzer

Filipe Sarmento

Flavia Machado

Flavia Nardes

Flávia Rolim

Flávio Augusto Sallem

Francisco Dias

Francisco Gondim

Francisco José Luccas

Francisco Luciano Pontes Junior

Gabriel André da Silva Mendes

Gabriel Marinheiro

Gabriela Cipolli

Gisele Silva

Gisella Gargiulo

Gislaine Baroni

Gloria Maria Tedrus

Gonzalo Forno

Guilherme Diogo Silva

Guilherme Mendonça

Gülfem Ezgi Ozaltin

Gustavo Franklin

Gustavo Lenci Marques

Gustavo Luvizutto

Gustavo Patriota

Hanna Tu

Haruki Koike

Hélio A. G. Teive

Henrique Ballalai Ferraz

Henrique Salmazo da Silva

Hinpetch Daungsupawong

Hsin Chien

Ingrid Faber

Isabela Starling

Isabelle Patriciá F. Soares Chariglione

Isadora de Oliveira Cavalcante

Jacy Parmera

Jaison Antonio Barreto

Janini Chen

Jano Alves Souza

Jefferson Becker

Jesse Di Giacomo

Jeyaraj Durai Pandian

Jiying Zhou

João Eudes Magalhães

João Andrade

Joge Correale

Jônatas de Oliveira

José Antonio Fiorot Jr

José Artur Costa D'Almeida

José Bragatti

Jose Francisco Manganelli Salomão

José Garbino

José Geraldo Speciali

José Luiz Pedroso

José Pedro Baima

José Schwartzman

Juliana Chichorro

Juliana Yokomizo

Kaikai Wang

Karina Oliveira

Karina Donis

Kelsey Potter-Baker

Kelson Almeida

Kette Dualibi Ramos Valente

Laiss Bertola

Laura Silveira-Moriyama

Lázaro Amaral

Leandro Barbosa

Leandro Iuamoto

Lécio Figueira Pinto

Léo Coutinho

Leonardo Caixeta

Leonardo Carbonera

Leonardo Cruz de Souza

Leonardo Galvão

Leonardo Rakauskas Zacharias

Leticia Januzi de A. Rocha

Leticia Sampaio

Liana Lisboa Fernandez

Lineu Werneck

Lisiane Seguti Ferreira

Livia Dutra

Lorena Rosa Santos de Almeida

Lucas Scardua

Lucia Iracema Zanotto Mendonça

Luciana Chiavegato

Luciana Ramalho Pimentel-Silva

Luciene Covolan

Lucio Huebra

Luis Henrique Castro Afonso

Luiz Augusto Andrade

Luiz Queiroz

Maira Barbosa

Manuela Fragomeni

Manuelina Brito

Maramelia Miranda

Marcela Câmara Machado Costa

Marcelo Caetano

Marcelo Cruzeiro

Marcelo de Brito

Márcio Alexandre Pena-Pereira

Marcio Nattan

Marco Antonio Araujo Leite

Marco Aurelio Fornazieri

Marco Lopes

Marco Pedatella

Marco Utiumi

Marcos Christiano Lange

Marcos Gomes de Freitas

Marcos Oliveira

Marcos RG freitas

Marcus Della Coletta

Marcus Vinícius Pinto

Maria Clara Zotin

Maria Fernanda Mendes

Maria Paula Foss

Maria Stefanatou

Mariana Spitz

Marianna de Moraes

Marilisa Guerreiro

Marina Alvim

Marina Farah

Mário Luciano de Mélo Silva Júnior

Mario Sato

Marlon Aliberti

Marlon Wycliff Caeira

Marlos Martins

Matheus Ballestero

Matheus Ferreira

Matheus Pedro

Mauro Eduardo Jurno

Mayara Machado

Michael Hornberger

Michel Satya Naslavsky

Michele Becker

Milena Sales Pitombeira

Millene Camilo

Ming Lv

Míriam Soares

Mirian Salvadori Bittar Guaranha

Muqaddas Masood

Mustafa Yilmaz

Natália Pessoa Rocha

Natalia Trombini Mendes

Nathália de Figueiredo

Nathane Braga

Nevin Kuloğlu Pazarcı

Nico Melzer

Nise Alessandra Sousa

Norberto Frota

Orlando Graziani Povoas Barsottini

Osvaldo Nascimento

Otto Fustes

Pablo Vinícius Silveira Feitoza

Panayiotis Patrikelis

Patricia Rzezak

Paulo Caramelli

Paulo Christo

Paulo José Lorenzoni

Paulo Kimaid

Paulo Mattos

Paulo Mei

Paulo Victor de Souza

Pedro André Kowacs

Pedro Braga-Neto

Pedro Brandão

Pedro Cougo

Pedro Fraiman

Pedro Melo Barbosa

Pedro Naves

Pedro Tomaselli

Priscila Klaes

Rachel Marin de Carvalho

Ramon Távora Viana

Raphael Casseb

Raphael Castilhos

Raphael Cirino

Raphael Spera

Regis Mestriner

Renan Castello Branco

Renan Domingues

Renata Barreto Tenório

Renata Kochhann

Renata Londero

Renata Sisto

Renata Viana Brigido de Moura Jucá

Renato Anghinah

Renato Puppi Munhoz

Ricardo Alvim

Ricardo Horta Maciel

Ricardo Nitrini

Rita Silveira

Roberta Diehl Rodriguez

Roberto Colasanti

Rodrigo Bazan

Rodrigo Brisson

Rogério Beato

Rogerio de Oliveira

Ronaldo Abraham

Rosa Salinas

Ruben J. Echemendia

Rubens José Gagliardi

Sakhawat Ali

Sanchita Sharma

Sanjeev Roy

Sarah Camargos

Selma Tekin

Sheila Trentin

Shuai Zhang

Silvia Stahl Merlin

Silvia Takada

Simone Barreto

Sonia Maria Azevedo Silva

Soniza Alves-Leon

Tallulah Spina Tensini

Tamine Capato

Tania Viel

Tarso Adoni

Taylor Zuleger

Thais Bento Lima da Silva

Thamires Magalhães

Thiago Silva

Thiago Vale

Timur Ekiz

Tissiana de Haes

Tomásia Frezatti

Turgay Demir

Vanderci Borges

Victor Calil

Victor Gadelha

Vinicius Montanaro

Vinicius Schoeps

Vitor Caldas

Vítor Tumas

Viviam Sanabria

Viviane Carvalho Crelier

wagner teixeira

Walter Arruda

Wanderley Bernardo

Wladimir Bocca Vieira de Rezende Pinto

Wyllians Borelli

Yang Tian

Yılmaz Inanc

Ylmar Correa-Neto

Yuai Han

